# Em Busca da Perfusão Coronariana Perfeita

**DOI:** 10.36660/abc.20210228

**Published:** 2021-05-06

**Affiliations:** 1 Universidade de Pernambuco RecifePE Brasil Universidade de Pernambuco, Recife, PE – Brasil.

**Keywords:** Síndrome Metabólica, Síndrome Coronariana Aguda, Infarto do Miocárdio, Perfusão do Miocárdio, Frequência Cardíaca, Acidente Vascular Cerebral, Lesão Renal, Prognóstico

Desde a faculdade, sempre ouvimos que “tempo é músculo”, e que quanto mais rápido reperfundimos uma artéria culpada por uma síndrome coronariana aguda (SCA), melhor para o paciente. Com o passar do tempo, a cardiologia baseada em evidências nos ensina que nem toda artéria aberta é igual. O fato de termos uma artéria com fluxo TIMI 3 parecia ser suficiente para definir o prognóstico do paciente. No entanto, após o conceito de isquemia microvascular, passamos a nos importar também com a perfusão dos pequenos vasos.^1^^,^^2^

Tendo em vista essa preocupação, surgiu o conceito de *no reflow*, que significa que mesmo após a recanalização de uma artéria culpada, haveria o risco do não restabelecimento do fluxo tecidual relacionado a aquele território miocárdico.^3^ Voltando mais no tempo, desde 1972 já havia sido descrito o fenômeno de *slow flow*,^4^ que é a lentificação da opacificação na ausência de doença coronariana obstrutiva epicárdica, mantendo a perfusão miocárdica. O *slow flow* parece ser mais comum em pacientes com síndrome metabólica, do sexo masculino e tabagistas.^5^ Tanto o fenômeno de *no reflow* quanto o de *slow flow* estão associados a desfechos cardiovasculares significativos; o primeiro caso está relacionado à disfunção ventricular e ao remodelamento cardíaco;^3^^,^^6^ e o segundo caso, às arritmias ventriculares ou à morte súbita,^7^^,^^8^ além de angina refratária.^9^

No trabalho do Dr. Huyut, publicado nesta edição de *Arquivos Brasileiros de Cardiologia*,^10^ encontramos uma nova abordagem sobre esse tema, na qual o autor procura comparar os dois fenômenos e as suas implicações clínicas, no contexto de uma SCA sem supradesnivelamento do segmento ST. Tanto do ponto de vista do “embate” entre essas duas entidades clínicas quanto por estarem sendo abordadas após um evento coronariano agudo, estamos diante de uma dissertação rara, talvez até inédita na literatura. Neste trabalho, um índice de massa corpórea acima de 28,3 kg/m^2^ e uma frequência cardíaca menor que 66,5 bpm foram preditores de *no reflow*. Os pacientes com este fenômeno tiveram maior incidência de acidente vascular encefálico e de eventos cardiovasculares maiores (MACE) ao final de um ano.^10^

Algumas limitações devem ser consideradas, como a discrepância entre os grupos analisados (221 pacientes com *slow flow versus* 25 com *no reflow*) e o fato de que não foi utilizada a ressonância nuclear magnética para avaliar a isquemia microvascular, o que seria o padrão-ouro para esse fim. No entanto, essas limitações não devem ofuscar a análise desse trabalho, que nos brinda, por outro lado, com um tempo de seguimento significativo (um ano) e desfechos clínicos importantes.^10^

Estamos falando de um tema que suscita ainda uma série de dúvidas. Por exemplo, como prevenir o *no reflow* nesses pacientes? Fármacos como os inibidores da glicoproteína IIb/IIIa podem ser recomendados para pacientes tempo porta-balão elevado, num contexto de SCA com supradesnivelamento do segmento ST. Será que isso vale na SCA sem supra? Dispositivos de prevenção de embolias em pacientes com lesões envolvendo enxertos venosos também tem sido recomendados,^11^ mas ainda é muito complicado avaliar quais indivíduos podem se beneficiar de qualquer estratégia de prevenção de *slow flow* ou *no reflow*, sobretudo diante de um contexto agudo. Será que existe alguma conduta preventiva realmente eficaz desses fenômenos, que apresentem desfechos clinicamente relevantes? Tratam-se de perguntas ainda sem resposta.

Em edição recente dos *Arquivos Brasileiros de Cardiologia*, o mesmo autor publicou a relação entre um marcador bioquímico (molécula-1 de lesão renal – KIM-1) e concluiu que os níveis séricos dessa e, observem, a frequência cardíaca mais baixa foram associados ao *no reflow* em pacientes com SCA e supradesnivelamento do segmento ST.^12^ Em contrapartida, estamos falando de um marcador ainda não disponível na prática clínica. O que temos para identificar os pacientes que irão desenvolver *no reflow/slow flow*, em termos práticos? Apenas a frequência cardíaca não me parece suficiente.

Outro trabalho também publicado no ABC em 2020, mostrou que os pacientes com fluxo coronariano lento (não relacionados a SCA) podem ter presença de realce tardio na ressonância nucelar magnética cardíaca e que nesses pacientes, o NT-proBNP parece estar mais elevado que no grupo controle,^13^ o que, em concordância com o trabalho apresentado aqui, mostra que esse fenômeno não tem nada de inofensivo.

Estamos ainda em um campo de muitas dúvidas nos pacientes que desenvolvem ou possuem algum tipo de disfunção microvascular, seja ela espontânea ou induzida por procedimento percutâneo. Contudo, o trabalho de Dr. Huyut lança alguma luz sobre esse terreno sombrio, enquanto nos estimula a seguir em busca da perfusão coronariana perfeita.
