# Supplementary material for: Em Busca da Perfusão Coronariana Perfeita
Source: Arq Bras Cardiol. 2021 May 6;116(5):865–6. [Article in Portuguese] doi: 10.36660/abc.20210228 (PMC8121469; doi:10.36660/abc.20210228)
Supplement: Translation

# Translation: In Search of the Perfect Coronary Perfusion

**Affiliations:** 1 RecifePE Brasil Universidade de Pernambuco, Recife, PE – Brasil.

**Keywords:** Metabolic Syndrome, Acute Coronary Syndrome, Myocardial Infarction, Myocardial Perfusion, Coronary Thrombosis, Heart Rate, Stroke, Kidney Injury Prognosis

Since medical school, we always heard that “time is muscle” and that the faster we reperfuse a culprit artery in acute coronary syndrome (ACS), the better for the patient. Over time, evidence-based cardiology has taught us that not every patent artery is the same. The fact that we have an artery with a “TIMI 3” flow seemed enough to define the patient’s prognosis, but after the concept of microvascular ischemia, we also started to care about small-vessel perfusion.^1^^,^^2^

Based on this concern, came the concept of ‘no reflow’, which means that even after the recanalization of a culprit artery, the tissue flow related to that myocardial territory might not be reestablished.^3^ Going back in time, the phenomenon of slow flow^4^ has been described since 1972, defined as delayed coronary opacification in the absence of epicardial obstructive coronary disease, while maintaining myocardial perfusion. Slow flow seems to be more common in patients with metabolic syndrome, in the male gender and smokers.^5^ Both the phenomenon of no reflow and slow flow are associated with significant cardiovascular outcomes, the first being related to ventricular dysfunction and cardiac remodeling^3^^,^^6^ and the latter, to cases of ventricular arrhythmias or sudden death,^7^^,^^8^ in addition to refractory angina.^9^

In the study by Dr. Huyut, published in this issue of the Brazilian Archives of Cardiology,^10^ we have a new approach on this topic, with the author trying to make a comparison between the 2 phenomena and their clinical implications, in the context of an ACS without ST-segment elevation. Both from the point of view of the “clash” between these two clinical entities, and because they are being approached after an acute coronary event, we are facing a rare dissertation, perhaps even unprecedented in the literature. In this study, a body mass index >28.3 kg/m^2^ and a heart rate below 66.5 bpm were predictors of no reflow, and patients with this phenomenon had a higher incidence of stroke and major adverse cardiovascular events (MACE) at the end of 1 year.^10^

Some limitations should be considered, such as the discrepancy between the analyzed groups (221 patients with slow flow vs. 25 with no reflow) and the fact that nuclear magnetic resonance was not used to assess microvascular ischemia, which would be the gold standard for that purpose. However, these limitations should not overshadow the analysis of this work, which, on the other hand, provides us with a significant time of follow-up (1 year) and with important clinical outcomes.^10^

We are talking about a topic that still raises many doubts. For instance, how to prevent no reflow in these patients? Drugs such as glycoprotein IIb/IIIa inhibitors may be recommended in patients with elevated door-to-balloon time in a context of ACS with ST-segment elevation, but does this apply to ACS without ST-segment elevation? Embolism prevention devices in patients with lesions involving venous grafts have also been recommended,^11^ but it is still very complicated to assess which patients can benefit from any slow flow or no reflow prevention strategy, especially in an acute context. And is there a preventive approach that is really effective for these phenomena, with clinically relevant outcomes? These questions remain unanswered.

In a recent edition of the Brazilian Archives of Cardiology, the same author published about the relationship between a biochemical marker (kidney injury molecule-1 - KIM-1) and found out that its serum levels and, there you are, a lower heart rate, were associated with no reflow in patients with ACS with ST-segment elevation.^12^ But we are talking about a marker not yet available in clinical practice. What do we have in clinical practice to identify patients who will develop no reflow/slow flow? The heart rate just does not seem to be enough.

Another study also published in ABC in 2020, showed that patients with slow coronary flow (not related to ACS) may have the presence of delayed enhancement on cardiac magnetic resonance imaging and that in these patients, NT-proBNP seems to be higher than in the group control,^13^ which, in agreement with the work presented here, shows that this phenomenon has nothing harmless.

We are still treading uncharted territory regarding patients who develop or have some type of microvascular dysfunction, whether spontaneous or induced by percutaneous procedure, but Dr. Huyut’s work sheds some light on this dark path, while encouraging us to continue in our search of the perfect coronary perfusion.
